# Postural threat increases sample entropy of postural control

**DOI:** 10.3389/fneur.2023.1179237

**Published:** 2023-06-05

**Authors:** Olivia M. Fischer, Kyle J. Missen, Craig D. Tokuno, Mark G. Carpenter, Allan L. Adkin

**Affiliations:** ^1^Department of Kinesiology, Brock University, St. Catharines, ON, Canada; ^2^School of Kinesiology, University of British Columbia, Vancouver, BC, Canada; ^3^Djavad Mowafaghian Centre for Brain Health, University of British Columbia, Vancouver, BC, Canada; ^4^International Collaboration on Repair Discoveries, University of British Columbia, Vancouver, BC, Canada

**Keywords:** postural control, balance, postural threat, sample entropy, attention focus, perceived anxiety, physiological arousal

## Abstract

**Introduction:**

Postural threat elicits modifications to standing balance. However, the underlying neural mechanism(s) responsible remain unclear. Shifts in attention focus including directing more attention to balance when threatened may contribute to the balance changes. Sample entropy, a measure of postural sway regularity with lower values reflecting less automatic and more conscious control of balance, may support attention to balance as a mechanism to explain threat-induced balance changes. The main objectives were to investigate the effects of postural threat on sample entropy, and the relationships between threat-induced changes in physiological arousal, perceived anxiety, attention focus, sample entropy, and traditional balance measures. A secondary objective was to explore if biological sex influenced these relationships.

**Methods:**

Healthy young adults (63 females, 42 males) stood quietly on a force plate without (No Threat) and with (Threat) the expectation of receiving a postural perturbation (i.e., forward/backward support surface translation). Mean electrodermal activity and anterior–posterior centre of pressure (COP) sample entropy, mean position, root mean square, mean power frequency, and power within low (0–0.05 Hz), medium (0.5–1.8 Hz), and high-frequency (1.8–5 Hz) components were calculated for each trial. Perceived anxiety and attention focus to balance, task objectives, threat-related stimuli, self-regulatory strategies, and task-irrelevant information were rated after each trial.

**Results and Discussion:**

Significant threat effects were observed for all measures, except low-frequency sway. Participants were more physiologically aroused, more anxious, and directed more attention to balance, task objectives, threat-related stimuli, and self-regulatory strategies, and less to task-irrelevant information in the Threat compared to No Threat condition. Participants also increased sample entropy, leaned further forward, and increased the amplitude and frequency of COP displacements, including medium and high-frequency sway, when threatened. Males and females responded in the same way when threatened, except males had significantly larger threat-induced increases in attention to balance and high-frequency sway. A combination of sex and threat-induced changes in physiological arousal, perceived anxiety, and attention focus accounted for threat-induced changes in specific traditional balance measures, but not sample entropy. Increased sample entropy when threatened may reflect a shift to more automatic control. Directing more conscious control to balance when threatened may act to constrain these threat-induced automatic changes to balance.

## 1. Introduction

Postural threat manipulations have been used to investigate the effects of emotions, such as fear and anxiety, on balance control (1). One common manipulation of postural threat involves altering the height of the support surface on which individuals stand. When tasked to quietly stand at or near the edge of an elevated platform, healthy young adults typically lean further away from the edge and adopt a balance strategy characterized by decreased amplitude and increased frequency of centre of pressure (COP) displacements in the anterior–posterior (A-P) direction (2–12). Postural threat has also been manipulated by having individuals quietly stand without or with the expectation of receiving an unexpected postural perturbation. When standing in anticipation of an A-P support surface translation, healthy young adults typically lean further forward and have increased amplitude and increased frequency of COP displacements in the A-P direction (13, 14). This research shows that threat-induced changes in leaning and sway amplitude vary with the threat context while increases in sway frequency specifically higher frequency components (>0.5 Hz) are consistent across these different types of postural threat (11, 12, 14), as well as other conditions of increased arousal and anxiety (15, 16).

Although threat-induced changes in balance are now well established, the underlying mechanism(s) that contribute to these changes remain poorly understood (1). Threat-induced changes in attention (e.g., directing more attention to balance) is one factor that may be responsible for the observed threat-induced balance changes. For example, healthy young adults report more conscious control of balance when standing on an elevated platform (7, 9) and direct more attention focus to balance, task objectives, threat-related stimuli, self-regulatory or coping strategies, and less attention focus to task irrelevant information in response to both surface height and postural perturbation threats (11–14, 17, 18). Concomitant reductions in attention to balance and high-frequency sway are observed when healthy young adults are repeatedly exposed to a surface height threat (11) and reductions in attention to self-regulatory/coping strategies and high-frequency sway are observed when standing in anticipation of a postural perturbation threat while performing a cognitive distractor task (14). Observed relationships between specific threat-induced changes in attention focus and balance further support attention as a mechanism underlying threat-induced balance changes. For example, healthy young adults who reported more conscious control of balance when standing on an elevated platform leaned further away from the edge (7). Furthermore, healthy young adults who had larger increases in attention to balance when threatened were more likely to show greater increases in sway frequency at height (17) and lean further forward and have a larger increase in sway amplitude in anticipation of a postural perturbation (13). Individuals who had a larger increase in attention to self-regulatory/coping strategies when threatened with a postural perturbation were more likely to have a larger increase in sway frequency (13).

Most studies examining the effects of postural threat on balance control have used traditional balance measures (e.g., amplitude- or frequency-based) to summarize the COP time-series. To further explore the possibility of attention underlying threat-induced changes in balance, the use of non-linear balance measures like sample entropy may provide novel insight into the balance strategy adopted in threatening conditions. Sample entropy assesses the regularity or predictability of COP time-series data and informs about the temporal dynamics or structure of the COP (19). The probability of a particular sequence of data points repeating itself in time is calculated; higher sample entropy values indicate a more irregular and unpredictable COP time-series (i.e., greater probability of observing different sequences in the data) while lower sample entropy values indicate a more regular and predictable COP time-series (i.e., greater probability of observing repeated sequences in the data) (20). Although a shift in sample entropy values (lower or higher) on a continuum may be interpreted differently (21), critical to the current study, these shifts are thought, by some, to reflect the attentional involvement in balance control (22, 23). In this case, higher sample entropy values are thought to reflect less attention to balance and a more automatic balance control; in contrast, lower sample entropy values are thought to reflect more attention to balance and a less effective control of balance. This perspective is supported by research that has shown lower sample entropy values in individuals with stroke compared to controls with sample entropy values shifting higher during stroke recovery as less attention to balance is needed (22). Lower sample entropy values have also been reported in individuals with vestibular deficits compared to controls (24). Higher sample entropy values have been reported in dance experts compared to non-dancers with the experts presumably needing to devote less attention to balance (25). A shift to lower sample entropy values has been shown when standing compared to sitting (23), when standing with eyes closed compared to eyes open (26), when standing with changes in the visual complexity of the environment (24), and when standing on a compliant compared to normal support surface (24, 27) with the more challenging task conditions thought to require a higher degree of attentional involvement in balance. In contrast, a shift to higher sample entropy values has been reported when standing and using external attention focus instructions (28, 29) or performing a concurrent cognitive task (30, 31) with these task constraints acting to direct attention away from standing promoting a more automatic control of balance (32). Research has also shown that individuals who report a greater tendency to consciously control movement have lower sample entropy values during a quiet standing task (33). In addition, studies that have directly manipulated conscious control of balance have revealed lower sample entropy values when individuals received movement monitoring instructions compared to when they were distracted from focusing on their balance (34, 35).

Based on this work, sample entropy or COP regularity is thought to reflect the amount of attention invested in postural control. Roerdink and colleagues suggested that postural threat would shift sample entropy values lower reflecting more attention to balance and less automatic behaviour (23). This view seems plausible as postural threat increases attention to balance (7, 9, 11–14, 17, 18). However, Stins and colleagues have reported no change in sample entropy when healthy young adults stood on a high compared to low platform (36). More recently, Ellmers and colleagues showed an increase in conscious motor processing that was accompanied by an increase in sample entropy suggesting a more automatic control when older adults stood on a high compared to low platform (37, 38), opposite to what would have been theoretically expected (23). Directing more conscious control to balance may constrain threat-induced automatic changes to balance; threat-induced increases in sample entropy were amplified when older adults were distracted in this threatening condition (37). Given these discrepancies, it is important to confirm how sample entropy changes in healthy young adults when threatened, and if these changes vary for a different type of postural threat manipulation.

The main objectives of this study were (1) to investigate the effects of postural threat on sample entropy, and (2) to explore the relationships between threat-related changes in physiological arousal, perceived anxiety, attention focus, sample entropy, and traditional balance control measures. Given prior observations of sex-dependent changes in balance when standing on an elevated surface (2), sex differences in autonomic responses to stress and anxiety (39–41), and the influence of personality traits on threat-induced changes in balance (9), a secondary objective of this study was to explore how biological sex may influence threat-related changes in physiological, psychological, attention focus, and balance responses. Understanding how other individual factors like biological sex influence these responses may have important implications for interpreting and addressing threat-induced changes in balance. Postural threat was manipulated by having healthy young adults stand with or without the expectation of receiving a postural perturbation allowing for a comparison between No Threat and Threat conditions. Data were taken from two published studies (13, 14) and combined to address these objectives. Apart from the larger data set that was created by combining the studies, a novel component of the current study was the investigation of sample entropy changes in response to the threat of a postural perturbation, which had not been examined in the previously published work.

As individuals report directing more attention to balance when threatened (7, 9, 11–14, 17, 18), a significant decrease in sample entropy was theoretically expected in the Threat compared to No Threat condition (23). It was also expected that a combination of biological sex and threat-induced changes in physiological, psychological, and attention focus measures would significantly predict threat-induced changes in sample entropy, with threat-induced changes in attention focus to balance emerging as the strongest predictor. For example, it was anticipated that larger increases in attention to balance would significantly account for larger decreases in sample entropy.

## 2. Materials and methods

Data was combined from two published studies that quantified threat-induced changes in physiological arousal, perceived anxiety, attention focus, and balance control measures (13, 14). The two studies used the same postural threat, standing with or without the expectation of receiving a postural perturbation. Although certain procedures differed between the two studies, there was always a no threat (i.e., one trial performed prior to any threat/perturbation experience) and threat (i.e., one trial performed after experience with the threat/perturbation) condition that formed the basis of the current analyses.

### 2.1. Participants

One-hundred and five healthy young adults (63 females, 42 males) were included in this study. Descriptive statistics for participant characteristics including trait measures of anxiety (State–Trait Anxiety Inventory) (42), movement reinvestment (Movement Specific Reinvestment Scale) (43, 44), and physical risk-taking (Domain-Specific Risk-Taking Scale, Recreational Domain) (45) are presented in Table 1. Details of these measures can be found in (9). Exclusion criteria were any self-reported neurological or musculoskeletal conditions that could influence balance control. All experimental procedures were performed in accordance with the Declaration of Helsinki and were approved by the Brock University Bioscience Research Ethics Board. Each participant provided written informed consent prior to the start of any experimental procedures.

**Table 1:** Mean and standard deviation (SD) values for participant characteristics.

	Females (n = 63) Mean (SD)	Males (n = 42) Mean (SD)	value of p
Age (years)	21.32 (2.60)	22.62 (3.05)	**0.021**
STAI (20–80)	37.71 (10.10)	35.21 (7.19)	0.169
MSRS-CMP (5–30)	19.14 (4.35)	19.07 (4.86)	0.937
MSRS-MSC (5–30)	16.59 (5.46)	14.81 (5.30)	0.101
DOSPERT (6–42)	23.24 (8.57)	26.45 (8.79)	0.065

Value of *p* from independent samples t-tests between females and males (bold font identifies a significant difference); STAI, state–trait anxiety inventory (20 items, 4-point Likert scale, range 20–80 with higher scores representing more trait anxiety) (42); MSRS, movement-specific reinvestment scale (43, 44); CMP, conscious motor processing subscale (5 items, 6-point Likert scale, range 5–30 with higher scores representing more trait CMP); MSC, movement self-consciousness subscale (5 items, 6-point Likert scale, range 5–30 with higher scores representing more MSC); DOSPERT, domain specific risk-taking scale (recreational domain only, 6 items, 7-point Likert scale, range 6–42 with higher scores representing more risk-tasking behaviour) (45).

### 2.2. Procedure

#### 2.2.1. Postural threat manipulation

Participants stood on a force plate (OR6-7, AMTI, Watertown, MA, United States) that was surrounded by a wooden platform (0.9 m x 1.6 m) fitted flush with its surface. The force plate and platform were secured to a motorized 4.3-m linear positioning stage (H2W Technologies Inc., Valencia, CA, United States). Participants were instructed to stand quietly with bare-feet, in a stance width equal to their foot length, with arms at their side, and their gaze fixed on an eye-level target located on the wall 4-m away. Stance width was kept consistent across all conditions by outlining with tape the position of the feet on the force plate. Throughout the experiment, a spotter was positioned beside the platform and participants wore a harness that was attached to a track secured to the ceiling.

Participants stood with no expectation of receiving a postural perturbation (No Threat) or with the expectation of receiving a postural perturbation (Threat). The perturbation was a temporally and directionally unpredictable support surface translation in the anterior or posterior direction (displacement = 0.25 m, peak velocity = 0.9 m/s, peak acceleration = 1.7 m/s^2^). No restrictions were placed on the use of balance recovery strategies.

#### 2.2.2. Experimental protocol

The following is the common protocol that participants experienced in the two studies (13, 14). First, participants completed one No Threat trial which served as a practice trial to address first trial effects on balance control (3, 46) and to prime the anxiety and attention focus questionnaires. Next, participants completed a second No Threat trial before continuing with the Threat trials. In each No Threat trial, participants stood with no expectation of receiving a postural perturbation. In each Threat trial, participants stood with the expectation of receiving a postural perturbation. The quiet stance duration prior to the delivery of the perturbation was varied to ensure the temporal unpredictability of the perturbation. The stance duration for one of these trials matched the stance duration for the No Threat trial. As such, No Threat (i.e., one trial performed prior to any threat/perturbation experience) and Threat (i.e., one trial performed after experience with the threat/perturbation) conditions with equal stance durations were used for comparison. The other Threat conditions were excluded from the analyses as they were only completed to give participants experience with the perturbation and to ensure the temporal unpredictability of the perturbation.

Table 2 shows the relevant No Threat and Threat conditions that 80 participants completed in the initial study (13) and 25 participants completed in the second study (14). In each study, participants experienced the same number of perturbations prior to the Threat condition that was used for comparison. Of note, a second No Threat trial was completed after the Threat trials in the initial study which confirmed the absence of any order effects (13). There were two additional blocks of No Threat and Threat trials completed while performing a secondary cognitive task in the second study (14). However, these blocks of trials always followed the first block of trials performed without the secondary cognitive task. The one noted difference between the studies was the quiet standing duration of the No Threat and Threat trials used for comparison (i.e., 30-s or 60-s). As stance duration can influence balance measures including sample entropy (47, 48), the decision was made to combine the data sets from the two studies and compare traditional balance measures and sample entropy between No Threat and Threat trials calculated over 30-s durations. Thus, only the first 30-s of the 60-s trials completed in the second study were used (14). To address any concern that changes in sample entropy may be due to a shorter time-series, sample entropy was also examined in 25 participants who completed 60-s of quiet standing in the No Threat and Threat trials (14). The same threat-induced increase in sample entropy was observed in this subset of participants. The results of this analysis are presented as Supplementary material Table 1. It should be noted that questionnaires for perceived anxiety and attention focus (described below) are, consequently, based on different durations of standing trials, but are unlikely to influence the outcomes of these measures.

**Table 2:** Experimental conditions used for the combined data set in the current study.

Threat condition	Expectation of perturbation	Quiet standing duration
Johnson et al. (13)		
**No Threat**	**No**	**30-s**
Threat*	Yes	30-s
Threat	Yes	10-s
Threat	Yes	15-s
**Threat**	**Yes**	**30-s**
No Threat	No	30-s
Johnson et al. (14)		
**No Threat**	**No**	**60-s**
Threat	Yes	5-s
Threat	Yes	30-s
**Threat**	**Yes**	**60-s**

No Threat and Threat conditions used for comparison in the current study are in bold font. These specific Threat conditions were selected as participants had previous experience with the perturbation. *Reflects a Threat trial in which no perturbation was delivered after the quiet standing period. Only the first 30-s of the 60-s quiet standing trials from (14) were used to standardize trial duration. Eighty participants were involved in (13) and 25 participants were involved in (14).

### 2.3. Dependent measures

#### 2.3.1. Physiological arousal

To estimate changes in physiological arousal and confirm that the perturbation threat generated a significant emotional response, electrodermal activity (EDA) was recorded using a constant voltage of 0.5 V to two silver–silver chloride (Ag/AgCl) electrodes (EL-507, BIOPAC Systems Inc., United States) placed on thenar and hypothenar eminences of the non-dominant hand (49). Prior to electrode placement, a skin preparation gel was applied to the palmar recording sites (NuPrep, Weaver and Company, United States). Electrodermal activity was A/D sampled at 1000 Hz (13) and down sampled to 100 Hz or sampled at 100 Hz ((14); Micro1401, CED, Cambridge, United Kingdom) and recorded using Spike2 software (CED, Cambridge, UK). A custom script that calculated mean EDA for the 30-s trial was used (MATLAB R2020a, MathWorks, United States).

#### 2.3.2. Perceived anxiety

Perceptions of anxiety were recorded from a self-report questionnaire to establish that the perturbation threat altered emotional state. The questionnaire was administered to evaluate worry-related and somatic anxiety. In the Johnson and colleagues (2019) study (13), responses were rated on a scale ranging from 0 (“I was not at all worried”) to 100 (“I was very worried”) on how respondents generally felt from the start to the end of the standing trial (or the time prior to platform translation). Responses to the question “How physically anxious did you feel when performing the balance task?” were rated on a scale ranging from 0 (“I did not feel anxious at all”) to 100 (“I felt very anxious”) to represent somatic anxiety. In the Johnson and colleagues (2020) study (14), these responses were rated on scales ranging from 1 to 9 with the same anchors. Thus, worry-related and somatic anxiety scores were converted to a percent of maximum possible score (50). Perceived anxiety was then calculated by averaging the scores of the worry-related and somatic anxiety questions.

#### 2.3.3. Attention focus

A questionnaire was administered to evaluate attention focus with the following statement preceding each question, “While completing the balance task, you may have directed your attention toward different information. Please indicate the extent to which you thought about or paid attention to: (1) movement processes (balance), (2) task objectives, (3) threat-related stimuli, (4) self-regulatory strategies, and (5) task-irrelevant information (17). Responses were rated on a 9-point Likert scale ranging from 1 (“Not at all”) to 9 (“Very much so”) on how respondents directed their attention from the start to the end of the standing trial, or the time prior to platform translation. This information was obtained to determine if there were broad changes in attention focus, and more specifically whether individuals reported more conscious control of balance when threatened.

#### 2.3.4. Sample entropy and traditional balance measures

Ground reaction forces and moments from the force plate were either sampled at 1000 Hz (13) and down sampled to 100 Hz or sampled at 100 Hz (14). All data was low-pass filtered offline using a second order Butterworth filter with a cut-off frequency of 10 Hz and used to calculate COP measures in the A-P direction (aligned with the direction of the postural threat). The COP measures from each trial were used to calculate summary measures of sample entropy and traditional balance measures, including mean position (COP-MPOS), root-mean-square (COP-RMS) amplitude, mean power frequency (COP-MPF) and the average power contained within specific frequency bands.

Sample entropy is the negative natural logarithm of the conditional probability that two similar sequences with the same amount of data points remain similar when another data point is added (19). Sample entropy in the A-P direction was calculated from customized MATLAB scripts [Mathworks, United States] presented by Richman and Moorman (19):


$$\mathrm{Sample Entropy}\;\left(m,r,N\right)=-\log\left(\;\frac{A}{B}\right)$$

where, *m* is the length of the sequences to be compared, *r* is the tolerance value for accepting matches, *N* is the length of the data, and *A/B* are defined as follows:


$$A=\left\{\frac{\left(n-m-1\right)\left(n-m\right)}{2}\right\}A^{m}\left(r\right)$$

, and


$$B=\left\{\frac{\left(n-m-1\right)\left(n-m\right)}{2}\right\}B^{m}\left(r\right)$$

where, A^m^(r) is the probability that sequences match for m + 1 points, and B^m^(r) is the probability that sequences match for m points. Parameter values were set to *m* = 2 and *r* = 0.15*SD. Although there is no established consensus on parameter selection, parameter settings for balance control studies are commonly set to *m* = 2 or 3, and *r* between 0.1 and 0.25*SD (19). Separate analyses calculated sample entropy in combinations of *m* = (2, 3) and *r* = (0.15, 0.25); sample entropy was consistent using these different parameter value combinations. Of note, sample entropy was calculated on the filtered COP data. As filtering has been shown to influence the calculation of sample entropy (51), sample entropy was also calculated on the unfiltered COP data to determine if the processing approach altered the effect of threat on this measure. While the absolute sample entropy values were higher when calculated from the unfiltered data, the directional effect of threat on sample entropy was the same. The results of these analyses are presented as Supplementary material Table 2.

Traditional balance measures, including mean position (COP-MPOS), root-mean-square (COP-RMS) amplitude, mean power frequency (COP-MPF) and the average power contained within specific frequency bands were also calculated from COP data. COP-MPOS was calculated to provide an estimate of leaning when referenced to participants’ ankle joints. COP-MPOS was subtracted from the COP signal to remove bias prior to calculating amplitude and frequency measures (52, 53). COP-RMS was used to provide a description of the COP time series magnitude. As a comprehensive assessment of postural control should involve several descriptors of the COP, including measures in both the time and frequency domains (54, 55), frequency-based measures were also calculated to provide information about the spectral properties of the COP which can inform about different processes involved in maintaining quiet stance. The Fast Fourier Transform was performed on equal length, non-overlapping data segments and converted to power spectra (56). Power spectrum analysis was used to estimate the average frequency contained within a power spectrum (COP-MPF) and the average power contained within specific frequency bands: 0–0.05 Hz (low frequency; COP-Freq_LOW_), 0.5–1.8 Hz (medium frequency; COP-Freq_MED_), and 1.8–5 Hz (high frequency; COP-Freq_HIGH_) (11, 57). All analyses were performed using MATLAB 2020a (Mathworks, United States).

### 2.4. Statistical analysis

#### 2.4.1. Repeated measures ANOVAs

Separate repeated measures analysis of variance (RM ANOVA) procedures with between-subject (biological sex; female, male) and within-subject (threat; No Threat, Threat) factors were performed for physiological arousal, perceived anxiety, attention focus, sample entropy, and traditional balance measures. The assumption of normality was confirmed prior to the statistical analysis. Non-normal variables (EDA, COP-RMS, COP-MPF, COP-Freq_LOW_, COP-Freq_MED_, COP-Freq_HIGH_) were corrected using logarithmic transformations, which calculated the base 10 logarithm of each value of the non-normal dependent variable. Significant biological sex by threat interaction effects were explored using Bonferroni-corrected *post hoc* tests. Significance level was set at *p* < 0.05.

#### 2.4.2. Multiple linear regressions

Change scores between Threat and No Threat conditions were calculated for each dependent variable. Multiple linear regressions were then conducted to determine if a combination of biological sex and threat-induced changes in physiological arousal, perceived anxiety, and attention focus measures contributed to explaining threat-induced changes in sample entropy and traditional balance measures. Bivariate correlations between biological sex, physiological, psychological, and attention focus change scores did not detect any significant collinearity; no variables were considered highly related (*r* > 0.80) and each of these variables were included as independent variables in the regressions. Seven multiple linear regressions were conducted with biological sex, physiological arousal, perceived anxiety, and attention to movement processes, task objectives, threat-related stimuli, self-regulatory strategies, and task-irrelevant information as the predictor variables and sample entropy, COP-MPOS, COP-RMS, COP-MPF, COP-Freq_LOW_, COP-Freq_MED_, and COP-Freq_HIGH_ as the dependent variables. Significance level was set at *p* < 0.05.

## 3. Results

### 3.1. Repeated measures ANOVAs

Descriptive statistics for physiological arousal, perceived anxiety, attention focus, sample entropy, and traditional balance measures for No Threat and Threat conditions for all participants and separately for females and males are presented in Table 3.

**Table 3:** Mean and standard error (SE) values for physiological, psychological, attention focus, sample entropy, and traditional balance measures for No Threat and Threat Conditions for all participants, females, and males, and RM ANOVA results.

	All Participants (*n* = 105)		Females (*n* = 63)		Males (*n* = 42)		RM ANOVA		
	No Threat Mean (SE)	Threat Mean (SE)	No Threat Mean (SE)	Threat Mean (SE)	No Threat Mean (SE)	Threat Mean (SE)	Sex value of *p*	Threat value of *p*	Interaction value of *p*
EDA (μS)	15.78 (0.63)	18.93 (0.71)	16.46 (0.86)	19.89 (0.98)	14.76 (1.04)	17.49 (1.15)	0.124	**<0.001**	0.296
ANX (%)	14.26 (1.84)	56.11 (2.59)	14.85 (2.55)	55.14 (3.49)	13.36 (2.59)	57.56 (3.85)	0.901	**<0.001**	0.465
AF-MP (1–9)	4.79 (0.24)	6.73 (0.20)	4.97 (0.30)	6.54 (0.27)	4.52 (0.40)	7.02 (0.29)	0.959	**<0.001**	**0.044**
AF-TO (1–9)	5.40 (0.23)	5.91 (0.22)	5.65 (0.29)	6.13 (0.30)	5.02 (0.36)	5.60 (0.31)	0.150	**0.016**	0.824
AF-TRS (1–9)	2.17 (0.14)	5.42 (0.23)	2.24 (0.19)	5.56 (0.29)	2.07 (0.21)	5.21 (0.35)	0.432	**<0.001**	0.684
AF-SRS (1–9)	3.56 (0.22)	4.77 (0.22)	3.87 (0.28)	5.44 (0.28)	3.10 (0.34)	3.76 (0.32)	0.113	**<0.001**	0.443
AF-TII (1–9)	3.60 (0.21)	2.29 (0.17)	3.75 (0.27)	2.51 (0.23)	3.38 (0.34)	1.95 (0.22)	0.156	**<0.001**	0.663
SampEn	0.093 (0.004)	0.131 (0.005)	0.088 (0.004)	0.124 (0.006)	0.100 (0.006)	0.142 (0.009)	**0.022**	**<0.001**	0.619
COP-MPOS (mm)	40.61 (1.97)	49.98 (2.09)	36.19 (2.54)	45.39 (2.77)	47.25 (2.84)	56.85 (2.86)	**0.004**	**<0.001**	0.881
COP-RMS (mm)	4.55 (0.17)	5.41 (0.20)	4.49 (0.22)	5.31 (0.26)	4.63 (0.29)	5.55 (0.32)	0.542	**<0.001**	0.823
COP-MPF (Hz)	0.26 (0.01)	0.41 (0.02)	0.25 (0.01)	0.38 (0.02)	0.28 (0.01)	0.45 (0.03)	**0.007**	**<0.001**	0.247
COP-Freq_LOW_ (mm^2^/bin)	99.61 (9.92)	114.76 (11.50)	98.93 (12.31)	111.98 (14.36)	100.64 (16.74)	118.94 (19.22)	0.795	0.280	0.856
COP-Freq_MED_ (mm^2^/bin)	0.65 (0.03)	2.09 (0.15)	0.62 (0.05)	1.89 (0.18)	0.69 (0.05)	2.39 (0.28)	0.096	**<0.001**	0.148
COP-Freq_HIGH_ (mm^2^/bin)	0.023 (0.002)	0.085 (0.008)	0.020 (0.002)	0.068 (0.007)	0.027 (0.003)	0.111 (0.015)	**0.003**	**<0.001**	**0.016**

Bold font value of *p*s identify significant differences. EDA, electrodermal activity; ANX, perceived anxiety; AF, attention focus; MP, movement processes; TO, task objectives; TRS, threat-related stimuli; SRS, self-regulatory strategies; TII, task-irrelevant information; SampEn, sample entropy; COP, centre of pressure; MPOS, mean position; RMS, root mean square; MPF, mean power frequency; Freq_LOW_, low frequency (0–0.05 Hz); Freq_MED_, medium frequency (0.5–1.8 Hz); Freq_HIGH_, high frequency (1.8–5 Hz).

#### 3.1.1. Threat effects

**Figure 1:**
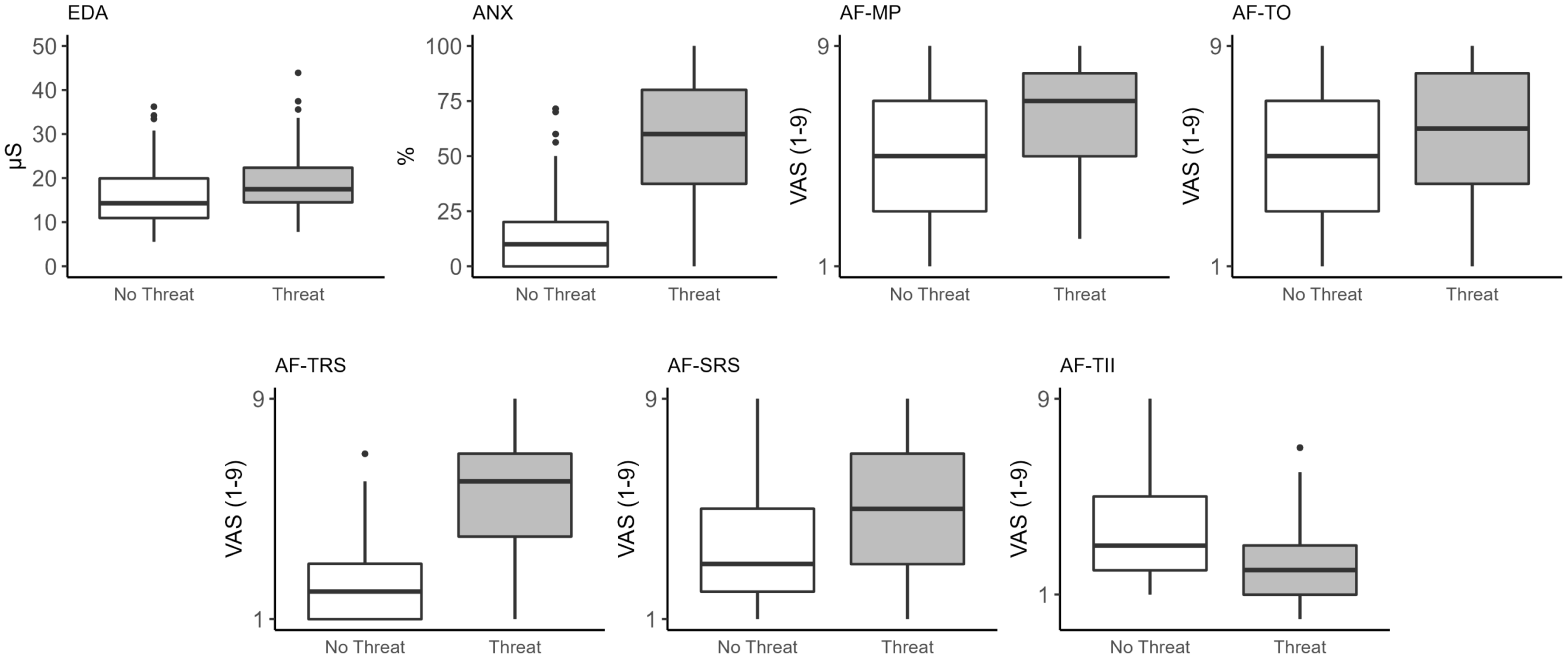
Box plots of the effects of threat on physiological, psychological, and attention focus measures. Significant differences were observed for all measures between No Threat and Threat conditions. EDA, electrodermal activity; ANX, perceived anxiety; AF, attention focus; MP, movement processes; TO, task objectives; TRS, threat-related stimuli; SRS, self-regulatory strategies; TII, task-irrelevant information.

A significant main effect of threat was observed for EDA (*F*_(1,103)_ = 87.93, *p* < 0.001) and perceived anxiety (*F*_(1,103)_ = 251.71, *p* < 0.001). Electrodermal activity was significantly greater, and participants reported more anxiety in the Threat compared to No Threat condition (Table 3; Figure 1). A significant main effect of threat was observed for attention to movement processes (*F*_(1,103)_ = 79.99, *p* < 0.001), task objectives (*F*_(1,103)_ = 5.99, *p* = 0.016), threat-related stimuli (*F*_(1,103)_ = 228.16, *p* < 0.001), self-regulatory strategies (*F*_(1,103)_ = 44.98, *p* < 0.001), and task-irrelevant information (*F*_(1,103)_ = 37.54, *p* < 0.001). Participants reported directing significantly more attention to movement processes, task objectives, threat-related stimuli, and self-regulatory strategies, and significantly less attention to task-irrelevant information (Table 3; Figure 1).

**Figure 2:**
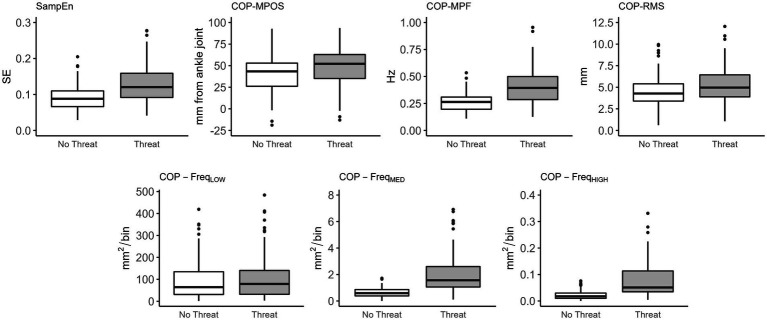
Box plots of the effects of threat on sample entropy and traditional balance measures. Significant differences were observed for all measures except for COP- Freq_LOW_ between No Threat and Threat conditions. SampEn, sample entropy; COP, centre of pressure; MPOS, mean position; RMS, root mean square; MPF, mean power frequency; Freq_LOW_, low frequency (0–0.05 Hz); Freq_MED_, medium frequency (0.5–1.8 Hz); Freq_HIGH_, high frequency (1.8–5 Hz).

A significant main effect of threat was observed for sample entropy (*F*_(1,103)_ = 39.86, *p* < 0.001). Sample entropy was significantly higher in the Threat compared to No Threat condition (Table 3; Figure 2). A significant main effect of threat was also observed for COP-MPOS (*F*_(1,103)_ = 50.82, *p* < 0.001), COP-RMS (*F*_(1,103)_ = 14.05, *p* < 0.001), and COP-MPF (*F*_(1,103)_ = 68.91, *p* < 0.001). Participants leaned significantly further forward and had significantly higher amplitude and frequency of COP displacements in the Threat compared to No Threat condition (Table 3; Figure 2). There was no significant main effect of threat for COP-Freq_LOW_ (*F*_(1,103)_ = 1.18, *p* = 0.280). However, a significant main effect of threat was observed for COP-Freq_MED_ (*F*_(1,103)_ = 104.67, *p* < 0.001) and COP-Freq_HIGH_ (*F*_(1,103)_ = 82.59, *p* < 0.001). COP-Freq_MED_ and COP-Freq_HIGH_ were significantly higher in the Threat compared to No Threat condition (Table 3; Figure 2).

#### 3.1.2. Biological sex effects

No main effects of biological sex were observed for EDA, perceived anxiety, or any of the five attention focus measures (Table 3). There were significant main effects of biological sex observed for sample entropy (*F*_(1,103)_ = 5.39, *p =* 0.022), COP-MPOS (F_(1,103)_ = 8.86, *p =* 0.004), COP-MPF (F_(1,103)_ = 7.66, *p =* 0.007) and COP-Freq_HIGH_ (F_(1,103)_ = 9.04, *p =* 0.003). Independent of threat, males compared to females had significantly higher sample entropy values (males, mean ± SE = 0.121 ± 0.008; females, mean ± SE = 0.106 ± 0.006), leaned further forward (males, mean ± SE = 52.05 ± 2.93 mm; females, mean ± SE = 40.79 ± 2.71 mm), had higher COP-MPF (males, mean ± SE = 0.36 ± 0.03 Hz; females, mean ± SE = 0.32 ± 0.02 Hz), and displayed higher COP-Freq_HIGH_ (males, mean ± SE = 0.069 ± 0.008 mm^2^/bin; females, mean ± SE = 0.044 ± 0.004 mm^2^/bin).

#### 3.1.3. Biological sex by threat interaction effects

There was a significant biological sex by threat interaction effect observed for attention to movement processes (*F*_(1,103)_ = 4.16, *p* = 0.044) that supersedes the main effect of threat observed for this measure. Both females and males directed significantly more attention to movement processes in the Threat compared to the No Threat condition (*p* < 0.001, for both groups) with the threat-induced change appearing to be larger for males (Table 3). However, there were no significant differences between females and males observed in the No Threat (*p* = 0.365) or Threat (*p* = 0.232) conditions.

There was also a significant biological sex by threat interaction effect observed for COP-Freq_HIGH_ (*F*_(1,103)_ = 6.01, *p* = 0.016) that supersedes the main effects of threat and biological sex observed for this measure. Follow-up comparisons revealed that COP-Freq_HIGH_ was significantly higher in the Threat compared to the No Threat condition for both females (*p* < 0.001) and males (*p* < 0.001) with the threat-induced change appearing to be larger for males (Table 3). There were also differences between females and males in the No Threat (*p* = 0.039) and Threat (*p* = 0.006) conditions.

No other significant biological sex by threat interaction effects were observed.

### 3.2. Multiple linear regressions

The multiple linear regression analyses revealed that a combination of biological sex and threat-induced changes in physiological arousal, perceived anxiety, and attention focus significantly accounted for changes in COP-RMS (*R*^2^ = 0.235, *F*_(8, 96)_ = 3.69, *p* < 0.001), COP-Freq_LOW_ (*R*^2^ = 0.158, *F*_(8, 96)_ = 2.25, *p* = 0.030), COP-Freq_MED_ (*R*^2^ = 0.227, *F*_(8, 96)_ = 3.53, *p* = 0.001), and COP-Freq_HIGH_ (*R*^2^ = 0.294, *F*_(8, 96)_ = 4.99, *p* < 0.001), but not sample entropy (*R*^2^ = 0.071, *F*_(8, 96)_ = 0.920, *p* = 0.504), COP-MPOS (*R*^2^ = 0.101, *F*_(8, 96)_ = 1.35, *p* = 0.228) or COP-MPF (*R*^2^ = 0.099, *F*_(8, 96)_ = 1.32, *p* = 0.245; Table 4). Significant predictors were biological sex, EDA, attention to movement processes, and attention to task-irrelevant information. Being male was associated with a larger increase in COP-Freq_HIGH_ (*β* = −0.217, *p* = 0.021) between Threat and No Threat conditions. A larger increase in EDA between Threat and No Threat conditions was significantly associated with a larger increase in COP-Freq_MED_ (*β* = 0.325, *p* < 0.001), and COP-Freq_HIGH_ (*β* = 0.298, *p* = 0.001). A larger increase in attention to movement processes between Threat and No Threat conditions was significantly associated with a larger increase in COP-RMS (*β* = 0.320, *p* = 0.005), COP-Freq_LOW_ (*β* = 0.304, *p* = 0.011) and COP-Freq_HIGH_ (*β* = 0.309, *p* = 0.005). A larger decrease in attention to task-irrelevant information was significantly associated with a larger increase in COP-Freq_MED_ (*β* = −0.209, *p* = 0.027).

**Table 4:** Multiple correlations (*R*^2^) and standardized beta weights for regressions between biological sex and threat-induced changes in physiological, psychological, and attention focus measures, and threat-induced changes in sample entropy and traditional balance measures.

	∆ SampEn	∆ COP-MPOS	∆ COP-RMS	∆ COP-MPF	∆ COP-Freq_LOW_	∆ COP-Freq_MED_	∆ COP-Freq_HIGH_
Sex	−0.106	0.000	0.044	−0.164	0.041	−0.152	**−0.217**
∆ EDA	0.109	0.102	0.075	0.230	0.006	**0.325**	**0.298**
∆ ANX	0.073	−0.115	0.132	−0.034	0.084	0.003	0.037
∆ AF-MP	−0.174	0.278	**0.320**	0.037	**0.304**	0.199	**0.309**
∆ AF-TO	−0.116	−0.024	0.110	−0.174	0.089	−0.072	−0.138
∆ AF-TRS	−0.153	−0.088	0.024	−0.009	−0.048	−0.019	0.018
∆ AF-SRS	0.110	0.144	0.020	0.155	0.067	0.130	0.091
∆ AF-TII	−0.003	−0.063	−0.073	−0.041	−0.061	**−0.209**	−0.145
*R^2^*	0.071	0.101	**0.235**	0.099	**0.158**	**0.227**	**0.294**
value of *p*	0.504	0.228	**<0.001**	0.245	**0.030**	**0.001**	**<0.001**

∆, measures represent change scores between Threat and No Threat conditions; EDA, electrodermal activity; ANX, perceived anxiety; AF, attention focus MP, movement processes; TO, task objectives; TRS, threat-related stimuli; SRS, self-regulatory strategies; TII, task-irrelevant information; SampEn, sample entropy; COP, centre of pressure; MPOS, mean position; RMS, root mean square; MPF, mean power frequency; Freq_LOW_, low frequency (0–0.05 Hz); Freq_MED_, medium frequency (0.5–1.8 Hz); Freq_HIGH_, high frequency (1.8–5 Hz). Bold font indicates a significant model or beta value.

## 4. Discussion

### 4.1. Effects of postural threat on physiological, psychological, and attention focus measures

The threat of a postural perturbation significantly altered physiological and psychological state in this group of healthy young adults. As anticipated, physiological arousal and perceptions of anxiety significantly increased when standing with the expectation of receiving a postural perturbation further revealing the efficacy of using this type of threat manipulation to study the effect of emotions on balance control (13, 14, 18, 58, 59). The threat of a postural perturbation also generated broad changes in attention focus. When threatened, participants increased attention towards balance, task objectives, threat-related stimuli, and self-regulatory or coping strategies, and decreased attention to task-irrelevant information. Similar changes in this threat-induced pattern of attention focus have been observed for surface height (11, 12, 17) and other postural perturbation threat (e.g., medial-lateral support surface translations) manipulations (18).

### 4.2. Effects of postural threat on sample entropy, and its relationships with physiological, psychological, and attention focus measures

A novel objective of this study was to examine the effects of postural threat on sample entropy, a measure of the regularity of the COP signal used to indicate the attentional involvement in balance control. A decrease in sample entropy is thought to reflect a shift to more attention needed to control balance and less automatic control, while an increase in sample entropy corresponds to less attention required for balance and more automatic control (22, 23). Given this interpretation, coupled with research that shows more attention directed to balance under conditions of postural threat (7, 9, 11–14, 17, 18), it was hypothesized that sample entropy would decrease when threatened. The results of the study revealed that postural threat did have a significant effect on sample entropy. However, this effect was opposite to that expected as an increase in sample entropy was observed when standing with compared to without the expectation of receiving a support surface perturbation. Interpreting this based on the assumptions underlying the sample entropy, attention, and automaticity relationship (22, 23), higher sample entropy values would suggest the use of a more automatic balance control strategy when threatened. Although this result is opposite to that theorized and incongruent with participants reporting more attention directed to their balance when threatened in this study, and other research that reveals greater cortical involvement in balance when threatened (60, 61), it does align with recent work that showed an increase in sample entropy in combination with increased conscious processing of balance when older adults experienced a surface height threat (37, 38). In this work, the authors suggested that the threat-induced increase in sample entropy occurs regardless of the threat-induced increase in conscious processing of balance. The increase in conscious processing of balance may serve to constrain automatic threat-induced balance changes acting as a strategy to limit the irregularity or unpredictability of the balance control system. Supporting this view, when participants were threatened and distracted from attending to their balance, sample entropy values continued to increase (37).

A second original aspect of the current study was exploring whether threat-induced alterations in physiological state, psychological state, and attention focus, including attention to balance, contributed to explaining threat-induced changes in sample entropy. The results of the multiple linear regression analysis showed that a combination of biological sex and threat-induced changes in physiological state, psychological state and attention focus did not predict threat-induced changes in sample entropy. These results did not confirm the hypothesis that attention focus and in particular attention to balance would be a significant predictor of changes in sample entropy and suggest that sample entropy may not be as susceptible to change through this mechanism under conditions of postural threat.

### 4.3. Interpretation of postural threat-induced changes in sample entropy

Despite the results of this study not supporting the theoretical assumptions of the sample entropy, attention and automaticity relationship, other possible explanations for the increase in sample entropy when standing with the expectation of receiving a postural perturbation need to be considered. It has been suggested that an increase or decrease in sample entropy can be interpreted in different ways (21). One explanation for the increase in sample entropy when threatened is that it reflects a heightened level of alertness and a shift to a more vigilant control of balance that prepares the system to deal with the threat of the unexpected postural perturbation. As physiological arousal and vigilance, although considered independent constructs, often vary together (62), it would be expected that changes in physiological arousal should be related to changes in sample entropy to support this interpretation. However, although physiological arousal (i.e., EDA) and sample entropy increased when threatened, threat-induced changes in physiological arousal were not associated with threat-induced changes in sample entropy.

A second explanation for the increase in sample entropy when threatened is that it may reveal an inability to use effective attention strategies. This interpretation may be supported by the threat-induced changes in attention focus to multiple sources beyond simply directing more attention to balance when threatened. This broad impairment in attention control (e.g., to balance, task objectives, threat-related stimuli, self-regulatory/coping strategies) may have produced greater interference when threatened, leading to the increase in sample entropy. Past research has used specific external and internal attention focus instructions or concurrent cognitive tasks to distract attention from balance to support the sample entropy, attention and automaticity relationship (28–31, 34, 35). If attention to balance had been the only change in attention reported by healthy young adults when threatened, the expected decrease in sample entropy may have been observed.

A third interpretation of the increase in sample entropy when threatened is that it may reflect greater noise present in the balance control system. This view may be supported by research that has revealed increased sensory gain in multiple sensory systems (e.g., proprioceptive, vestibular) (59, 63–68) and increased cortical excitability (69, 70) in response to a surface height threat.

At this point, the results of the current study are not able to definitively support one of these interpretations. As research has shown that threat-induced changes in balance are not always aligned with changes in attention or perceptions of sway (10), it is likely that a combination of attentional and neurophysiological mechanisms combine to influence the postural control strategy used when threatened (1).

### 4.4. Effects of postural threat on traditional balance measures

The balance strategy as described using traditional COP summary measures was also significantly different when standing with compared to without the expectation of receiving a postural perturbation. Healthy young adults leaned further forward and demonstrated increased amplitude and frequency of COP displacements, specifically in the higher frequency bands (i.e., > 0.5 Hz) when threatened. Increased amplitude and frequency of COP displacements in the medial-lateral direction have also been observed in response to the threat of a medial-lateral support surface translation (18). The findings from the current study reinforce but also expand upon the results reported in the two published studies from which the data set for this study was derived (13, 14). For example, inconsistencies in threat-induced changes in amplitude of COP displacements (i.e., increased or no change) between studies were resolved and threat-induced increases in the higher frequency components of sway which contribute to the increase in MPF across studies were confirmed in the larger sample used in the present study.

Past research has revealed inconsistent relationships between threat-induced changes in physiological state, psychological state, attention focus, and traditional balance measures (7, 13, 17). Therefore, it was important to examine these relationships in a larger sample to potentially inform about the mechanisms underlying threat-induced changes in balance. Combining the data from two previously published studies revealed that although biological sex, physiological state, psychological state, and attention focus measures were not related to sample entropy, they were related to specific traditional balance measures. The multiple linear regressions showed that a combination of these measures could predict amplitude of sway, and the low, medium, and high-frequency components of sway. Physiological arousal and attention to balance emerged as the most common significant predictors. A larger increase in attention to balance was associated with leaning further forward and a larger increase in low and high-frequency sway, while a larger increase in EDA was associated with a larger increase in medium and high-frequency components of sway. These relationships are different from these reported by Johnson and colleagues who reported larger increases in attention to balance being associated with leaning further forward and having larger increases in amplitude of sway, while larger increases in attention to self-regulatory/coping strategies were associated with larger increases in sway frequency (i.e., MPF) (13). Taken together, these results partially support the work of Ellmers and colleagues who showed parallel increases in conscious motor processing, sample entropy and higher frequency components of sway when older adults faced a surface height threat manipulation (37).

### 4.5. Sex differences in threat-induced behaviour

A secondary aim of this study was to explore how biological sex interacted with threat-induced changes in, and associations between, psychological, physiological, attention focus and balance responses. Females and males responded in much the same way when standing with the expectation of a receiving a postural perturbation. Threat-induced changes in physiological arousal and perceived anxiety were not influenced by sex. Although previous research has revealed sex-differences in autonomic responses to stress and anxiety (39–41), these sex differences did not emerge in the current study. Although there were some sex differences in balance control that were found independent of threat, only attention focus to balance and high-frequency sway measures revealed a significant interaction between sex and threat, and sex only emerged as a significant predictor along with attention to balance and physiological arousal for high-frequency sway. In general, it appears that only the magnitude but not the direction of the threat-induced change was different between females and males, with males having a larger change in attention to balance and high-frequency sway when threatened. Previous sex differences in balance control in response to a surface height threat have been observed. However, these findings were different from the current results as females compared to males demonstrated a larger increase in MPF when standing on a high compared to low surface height (2).

Despite sex not significantly interacting with many threat-related changes in psychological, physiological, attention focus and balance responses and not emerging as a significant predictor for these threat-related changes, future work should be directed to identify if a combination of sex and personality traits (e.g., trait anxiety, movement reinvestment, and risk taking), although not significantly different between males and females in this study, can explain threat-induced behaviour.

### 4.6. Limitations

The results of this study are only generalizable to healthy young adults experiencing a postural perturbation threat. It is unknown if changes in sample entropy and relationships between sample entropy and other physiological, psychological, attention focus, and balance measures may differ under different threat contexts or in different populations (e.g., older adults reporting a fear of falling). The unbalanced number of females and males in this study may have also been a limitation for observing sex differences. Another possible limitation was that the COP time-series data from the Johnson and colleagues (2020) study (14) was shortened to 30-s so as to combine the data with that from the Johnson and colleagues (2019) study (13). Although this allowed for consistency in time-series length when comparing traditional balance measures and sample entropy, it has been recommended to use at least time-series of 60-s for calculating traditional balance measures (47) and sample entropy (48).

## 5. Conclusion

A robust emotional response, as evidenced by increases in physiological arousal and perceived anxiety, and more conscious control of balance, were observed when standing with compared to without the threat of a postural perturbation. Sample entropy and high-frequency postural sway increased when threatened suggesting a shift to a more automatic control strategy. Given the theoretical assumptions underlying the interpretation of sample entropy, higher sample entropy values are typically associated with less attention to balance (22, 23). Although the current findings are incongruent with this expected relationship, directing more conscious control to balance when threatened may act to constrain these threat-induced automatic changes to balance (37). However, given the evidence of broad threat-related changes in attention focus (i.e., shifts in attention focus to multiple sources), this increase in sample entropy may also be interpreted as an inability to employ effective attention control in this threatening context (21). As past research has also revealed changes in sensory and cortical processing when threatened (1), an increase in sample entropy may reflect increased noise in the balance control system. It is likely that the effects of threat on balance control rely on a complex interaction between changes in attentional and neurophysiological processes (1). Future work should be directed to investigating complementary traditional and non-linear balance measures to inform about the potential mechanisms underlying changes in balance under different threat scenarios.

## Data availability statement

The raw data supporting the conclusions of this article will be made available by the authors, without undue reservation.

## Ethics statement

The studies involving human participants were reviewed and approved by Brock University Bioscience Research Ethics Board. The patients/participants provided their written informed consent to participate in this study.

## Author contributions

OF, KM, CT, MC, and AA contributed to the conception and design of the study. OF and KM organized and analyzed the data. AA and MC contributed to the first draft of the manuscript. All authors contributed to the article and approved the submitted version.

## Funding

This work was supported by Natural Sciences and Engineering Research Council of Canada (NSERC) grants to CT (386609), MC (326910), and AA (288164). Parts of this work were reported in an author’s Master’s thesis [OF (2021). The effects of postural threat on sample entropy. Master’s thesis. Brock University, http://hdl.handle.net/10464/15157].

## Conflict of interest

The authors declare that the research was conducted in the absence of any commercial or financial relationships that could be construed as a potential conflict of interest.

## Publisher’s note

All claims expressed in this article are solely those of the authors and do not necessarily represent those of their affiliated organizations, or those of the publisher, the editors and the reviewers. Any product that may be evaluated in this article, or claim that may be made by its manufacturer, is not guaranteed or endorsed by the publisher.
