# Supplementary material for: Postural threat increases sample entropy of postural control
Source: Front Neurol. 2023 Jun 5;14:1179237. doi: 10.3389/fneur.2023.1179237 (PMC10277644; doi:10.3389/fneur.2023.1179237)
Supplement: Supplementary file 1 [file Table_1.docx]

Supplementary Material

Table 1. Comparison of threat effects on balance outcomes between 30s and 60s for only the 25 participants from the Johnson et al. (2020) study. Values represent mean and standard error.

|  | 30s Duration |  |  | 60s Duration |  |  |
| --- | --- | --- | --- | --- | --- | --- |
|  | No Threat | Threat | p-value | No Threat | Threat | p-value |
| SampEn | 0.091 ± 0.008 | 0.115 ± 0.008 | 0.059 | 0.072 ± 0.005 | 0.095 ± 0.006 | 0.002 |
| COP-MPOS (mm) | 17.40 ± 3.10 | 21.22 ± 3.37 | 0.005 | 17.29 ± 3.27 | 21.73 ± 3.41 | 0.002 |
| COP-RMS (mm) | 3.55 ± 0.22 | 4.10 ± 0.35 | 0.103 | 4.07 ± 0.26 | 4.31 ± 0.29 | 0.319 |
| COP-MPF (Hz) | 0.29 ± 0.02 | 0.37 ± 0.02 | 0.026 | 0.21 ± 0.01 | 0.27 ± 0.02 | 0.005 |
| COP-FreqLOW (mm2/bin) | 48.46 ± 8.56 | 79.58 ± 23.55 | 0.156 | 91.31 ± 15.82 | 98.68 ± 18.17 | 0.656 |
| COP-FreqMED (mm2/bin) | 0.61 ± 0.09 | 0.99 ± 0.13 | 0.028 | 0.54 ± 0.07 | 0.88 ± 0.10 | 0.007 |
| COP-FreqHIGH (mm2/bin) | 0.017 ± 0.003 | 0.034 ± 0.006 | 0.009 | 0.012 ± 0.002 | 0.027 ± 0.004 | 0.002 |
