# Supplementary material for: Postural threat increases sample entropy of postural control
Source: Front Neurol. 2023 Jun 5;14:1179237. doi: 10.3389/fneur.2023.1179237 (PMC10277644; doi:10.3389/fneur.2023.1179237)
Supplement: Supplementary file 2 [file Table_2.docx]

Supplementary Material

Table 2. Sensitivity analysis of sample entropy parameters m and r with unfiltered and filtered data for all participants. Values represent mean and standard error.

|  |  | M = 2 |  |  | M = 3 |  |  |
| --- | --- | --- | --- | --- | --- | --- | --- |
|  |  | No Threat | Threat | p-value | No Threat | Threat | p-value |
| R = 0.15 | Unfiltered | 0.119 ± 0.006 | 0.151 ± 0.006 | <0.001 | 0.118 ± 0.006 | 0.150 ± 0.006 | <0.001 |
|  | Filtered | 0.093 ± 0.004 | 0.131 ± 0.005 | <0.001 | 0.097 ± 0.004 | 0.133 ± 0.005 | <0.001 |
| R = 0.25 | Unfiltered | 0.064 ± 0.003 | 0.083 ± 0.003 | <0.001 | 0.062 ± 0.003 | 0.084 ± 0.003 | <0.001 |
|  | Filtered | 0.052 ± 0.002 | 0.074 ± 0.003 | <0.001 | 0.053 ± 0.002 | 0.077 ± 0.003 | <0.001 |
